# Do Sugar-Sweetened Beverages Increase Fasting FGF21 Irrespective of the Type of Added Sugar? A Secondary Exploratory Analysis of a Randomized Controlled Trial

**DOI:** 10.3390/nu14194169

**Published:** 2022-10-07

**Authors:** Bettina Geidl-Flueck, Michel Hochuli, Giatgen A. Spinas, Philipp A. Gerber

**Affiliations:** 1 Department of Endocrinology, Diabetology and Clinical Nutrition, University Hospital Zurich (USZ), 8091 Zurich and University of Zurich (UZH), 8006 Zurich, Switzerland; 2 Department of Diabetes, Endocrinology, Nutritional Medicine and Metabolism, Inselspital, Bern University Hospital and University of Bern, 3010 Bern, Switzerland

**Keywords:** glucose, fructose, sucrose, sugar-sweetened beverage, FGF21, liver, homeostasis, healthy men, randomized controlled trial

## Abstract

Human fibroblast growth factor 21 (FGF21) is a multifaceted metabolic regulator considered to control sugar intake and to exert beneficial effects on glucose and lipid metabolism. Elevated serum FGF21 levels are associated with metabolic syndrome, suggesting a state of FGF21 resistance. Further, given the evidence of a hepatic ChREBP and FGF21 signaling axis, it can be assumed that SSBs containing fructose would possibly increase FGF21 concentrations. We investigated the effects of sugar-sweetened beverage (SSB) consumption on fasting FGF21 levels in healthy, lean men, discriminating the effects of glucose, fructose, and their disaccharide sucrose by secondary data analysis from a randomized controlled trial. Seven weeks of daily SSB consumption resulted in increased fasting FGF21 in healthy, lean men, irrespective of the sugar type. Medians of ΔFGF21 between post-SSB intervention values (week 7) and no-intervention period values (IQR) in pg/mL were: glucose 17.4 (0.4–45.8), fructose 22.9 (−8.6–35.1), and sucrose 13.7 (2.2–46.1). In contrast, this change in FGF21 concentration was only 6.3 (−20.1–26.9) pg/mL in the control group. The lack of a fructose-specific effect on FGF21 concentrations is contrary to our assumption. It is concluded that SSB intake may impact FGF21 concentrations and could contribute to the increased FGF21 concentrations observed in subjects suffering from metabolic syndrome that is possibly associated with decreased FGF21 responsiveness.

## 1. Introduction

For many people, daily consumption of sugar-sweetened beverages (SSBs) is an inherent part of their diet. High free-sugar intake (e.g. in the form of beverages) has been identified as a factor promoting metabolic alterations that may not only lead to the development of obesity [1], but also type 2 diabetes [2], non-alcoholic fatty liver disease (NAFLD) [3], cardiovascular disease [4] and other complications. In particular, previous studies in healthy men revealed alterations such as decreased hepatic insulin sensitivity [5], increased hepatic lipogenic activity/lipogenesis [6,7] and atherogenic lipid profiles [8] induced by regular consumption of SSBs containing fructose. In the present work, we investigated the effect of SSB consumption on fibroblast growth factor 21 (FGF21), which is considered a liver-derived hormone with multifaceted acute and chronic effects on metabolism [9] and which impacts the regulation of food intake. In particular, it regulates simple sugar intake and sweet taste preference [10], stimulates thermogenesis/energy expenditure [11] and has beneficial effects on glucose [12] and lipid metabolism [13]. FGF21 is supposed to have therapeutic potential for the treatment of metabolic syndrome and obesity, as it increases glucose uptake by the muscles [14] and adipose tissue (AT), induces adiponectin secretion by AT [15] and increases energy expenditure due to induction of browning of white adipose tissue (WAT) [16,17]. Paradoxically, serum FGF21 levels are elevated in subjects with obesity [18,19], metabolic syndrome [18], NAFLD [20] and coronary artery/heart disease [21,22]. It is hypothesized that increased FGF21 levels could indicate a state of FGF21 resistance [23,24].

FGF21 is subject to a complex regulatory network. A variety of stimuli have been found to induce FG21 expression in various tissues [25]. However, the major origin of circulating FGF21 is reported to be the liver [9]. Macronutrient intake is an important regulator of FGF21 levels, and its effects on FGF21 have been explored in numerous animal and human studies. While studies in mice have shown that starvation and ketogenic diets increase FGF21 serum concentrations [26,27], these effects did not occur in human dietary studies, indicating differences between species [19]. In contrast, both rodent and human studies demonstrated that protein restriction increases FGF21 levels [28]. Studies in humans showed that FGF21 is transiently increased after a glucose, fructose or sucrose challenge [29,30,31], with fructose inducing an earlier and stronger FGF21 response compared to glucose (75 g challenge). The FGF21 response after a fructose or sucrose (disaccharide of glucose and fructose) challenge are in a similar range [30]. Interestingly, the serum FGF21 response to simple sugar intake is enhanced in subjects with metabolic syndrome compared to healthy subjects [30].

While acute effects of simple sugar intake on FGF21 serum concentrations are known, there is a gap of knowledge regarding the impact on FGF21 levels of repeated SSB consumption in moderate amounts. This study aimed to describe the effects on fasting FGF21 serum concentrations of daily SSB intake over a period of seven weeks in healthy, lean men by a secondary, descriptive evaluation of data from a randomized controlled trial. In particular, it focuses on reporting the effects of different sugars, such as glucose, fructose and the disaccharide sucrose.

We supposed that prolonged exposure to fructose- and sucrose-containing SSBs would result in higher FGF21 levels, since it has been suggested that FGF21 secretion is mediated by ChREBP, which itself is strongly induced by fructose. In addition, the study also describes the changes in food intake resulting from SSB consumption (macronutrient composition and energy intake).

## 2. Methods

### 2.1. Study Design

A randomized controlled SSB trial was conducted in the years 2013–2016 at the University Hospital of Zurich to investigate the effects of SSB consumption on hepatic de novo lipogenesis (primary trial) [6]. The present study is based on data from this trial and includes additional exploratory analyses of the trial data. Over the course of 7 weeks, subjects consumed daily fructose-, sucrose- or glucose-sweetened beverages (80 g sugar/day) or had to abstain from SSB consumption (control). Subjects were randomly assigned to one of four dietary intervention groups (simple random allocation) and supplied with 2 dl SSB containers (13.3 g sugar/dl) containing pure fructose, sucrose or glucose, or no SSBs (control) (Molkerei Biedermann AG, Bischofszell (provided SSBs in coded containers); Swiss technology testing service, Dietikon (quality control)). These SSBs were produced exclusively for the trial and not commercially available. The drinks had to be consumed with the three main meals during the day.

### 2.2. Subjects

A total of 126 healthy male volunteers (age 18–30 years) with BMI < 24 kg/m^2^ were recruited to the study (primary trial) by advertisement at the University of Zurich in the years 2013–2016. Study participation was limited to only one sex (males), as there is evidence of divergent metabolic effects of fructose on males and females [32]. Eligibility was assessed by examination and included medical history and blood biochemistry. Subjects with high SSB consumption (exceeding 3 dl/day) or engaging in more than 3 h of physical activity per week were excluded from the study.

The study was approved by the Ethical Committee (Canton Zurich, Switzerland). Written informed consent was obtained from all individuals, and all procedures were performed in compliance with the guidelines of the Declaration of Helsinki. A total of 83 subjects gave consent for further use of their data and blood samples for secondary analyses and thus were included in the present study.

### 2.3. Protocol

At baseline and after seven weeks of intervention, subjects were examined at the Clinical Trial Unit, University Hospital Zurich as described in [6]. After an overnight fast, routine anthropometric data were collected (weight, height, waist and hip circumference) and blood samples were drawn for the measurement of laboratory parameters. On both occasions, the participants provided food records of their dietary intake for the three days before the examination.

### 2.4. Anthropometry

Subjects were weighed using a digital balance (WB 100 P, Tanita, Hoofddorp, the Netherlands), and height was measured using a wall-mounted stadiometer. Waist and hip circumference were determined using a measuring tape.

### 2.5. Laboratory Analysis

Blood glucose was measured from whole blood samples (BIOSEN C-line, EKF Diagnostic, Barleben, Germany). Triglycerides (TG), cholesterol and free fatty acids (FFA) were measured enzymatically in fresh serum (triglycerides, GPO-PAP; cholesterol, CHOP-PAP; HDL-cholesterol plus 3rd generation, Roche Diagnostics, Mannheim, Germany; free fatty acids, Konelab Free Fatty Acids; Thermo Scientific, Dreieich, Germany). From frozen serum, C-peptide was measured using an immunoradiometric assay (IRMA-C-PEP; CIS bio international, Bagnols-sur-Cèze Cedex, France), insulin was measured by radioimmunoassay (RIA; CIS Bio international, Oris Industries, Gif-Sur-Yvette, France) and leptin, adiponectin and FGF21 were measured using ELISA (Leptin EZHL-80 SK; Linco Research, St. Charles, MS, USA; adiponectin DRP 300, R&D Systems Inc., Minneapolis, MN, USA, ELISA; human FGF21-ELISA, RD191108200R, Brno, Czech Republic).

### 2.6. Dietary Assessment

For assessment of dietary intake, subjects recorded their food and beverage intake in a 3-day diary (weighed food record) [33]. A detailed analysis of the dietary intake was performed using a nutrition software system (EBISpro for Windows 8.0 (Swiss version), Dr J. Erhardt, University of Hohenheim, Hohenheim, Germany) that converts the reported consumed food into individual nutrients.

### 2.7. Statistics

Statistical calculations were performed with SPSS version 26 (IBM). As this is a purely exploratory analysis, we show descriptive statistics only. All variables and differences were tested for normal distribution. Accordingly, data are expressed as means with confidence intervals or medians with interquartile ranges (IQR) (non-normally distributed data in at least one group).

## 3. Results

### 3.1. Anthropometry

Data of 83 subjects (mean age 22.9 ± 2.5 years) were included in the analyses. Baseline anthropometric characteristics are reported in Table 1. Overall, the SSB intervention did not change anthropometric characteristics.

### 3.2. Glucose and Lipid Metabolism

Parameters of glucose and lipid metabolism are reported in Table 2. Fasting glucose and insulin values were comparable before and after the intervention, while C-peptide tended to be increased after the sucrose intervention (mean Δ (CI) 55.8 (6.0–105.6) pmol/L).

### 3.3. Fasting FGF21 Concentrations

**Figure 1:**
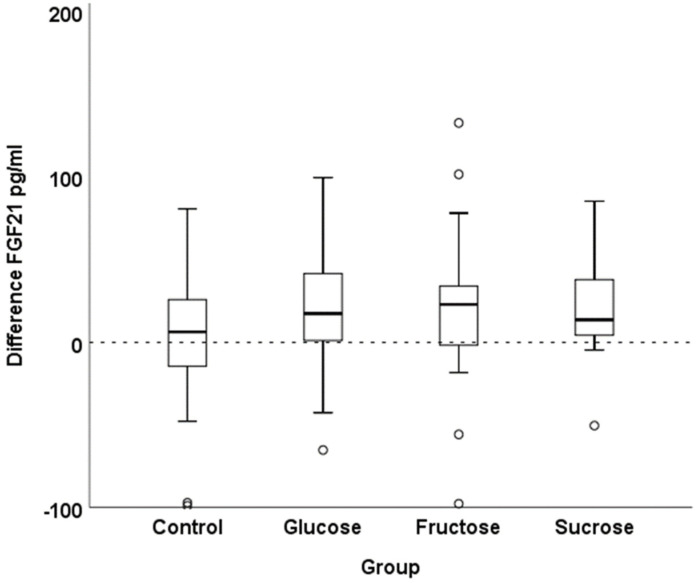
Difference in FGF21 concentrations between post-SSB intervention values or SSB abstinence values (control) (week 7), respectively, and baseline values (week 0) (boxplot with medians).

Fasting FGF21 concentrations showed some variability between subjects (Table 2). The increase after 7 weeks in the SSB groups (as compared to baseline) was higher than in the control group. Medians of differences of the SSB intervention groups were 2.2- (sucrose), 2.8- (glucose) and 3.6- (fructose) fold higher relative to the control group. Figure 1 illustrates the differences in FGF21 concentrations between baseline and after 7 weeks of SSB intervention.

### 3.4. Food Intake

**Figure 2:**
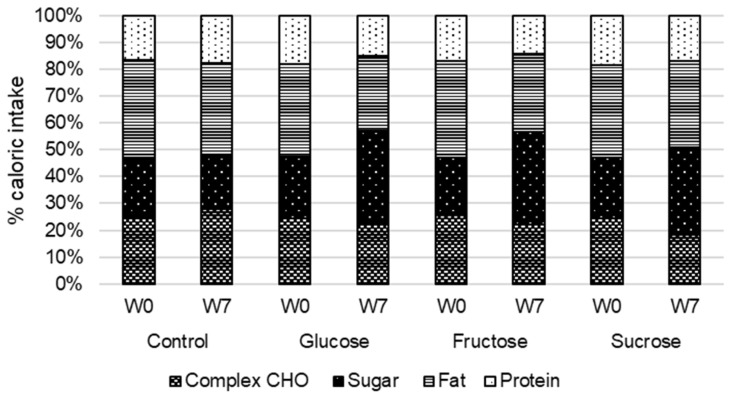
Macronutrient composition of the diet at baseline (W0) and at week 7 (W7) of the intervention.

The dietary intakes at baseline and after 7 weeks of SSB intervention are shown in Table 3 and Figure 2. There is some variability regarding the baseline sucrose intake. The intake of the different sugars changed according to the specific SSB interventions, and the total sugar consumption increased accordingly. Overall, there was no apparent increase in total energy intake by SSB interventions.

SSB interventions changed macronutrient ratios. The percentage of energy intake from carbohydrates increased in all SSB interventions by 4 to 11 percent. SSB intervention resulted in decreased fat intake in the glucose group and fructose group by 4–7 percent. SSB intervention also reduced protein intake by 2–3 percent.

### 3.5. Adipokines

**Figure 3:**
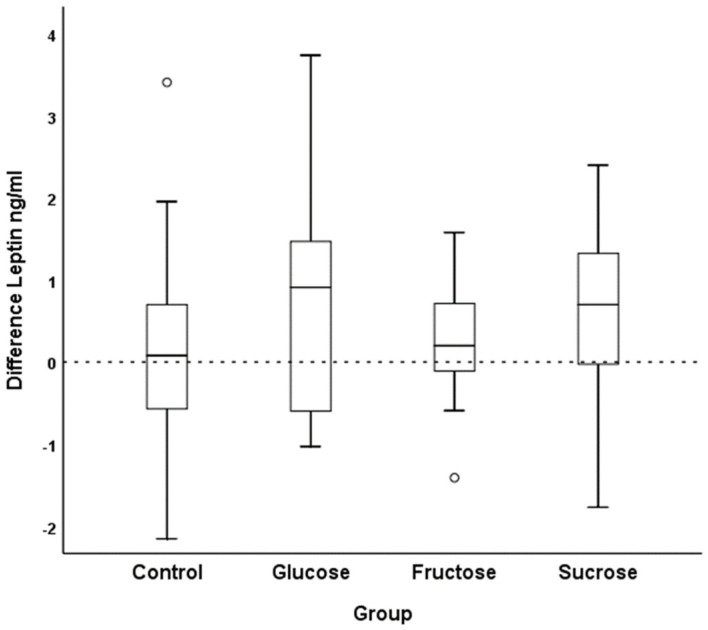
Difference in leptin concentrations between post-SSB intervention values or SSB abstinence values (control) (week 7), respectively, and baseline values (week 0) (boxplot with medians).

Adipokine concentrations are reported in Table 2. Leptin levels tended to be higher after the glucose (mean Δ (CI) 0.6 (−0.2–1.4) ng/mL) and sucrose interventions (0.6 (0.1–1.1) ng/mL) (Figure 3 illustrates the distribution of differences). In contrast, changes in levels of resistin and adiponectin were comparable throughout the groups.

## 4. Discussion

This secondary descriptive reporting of data from a subgroup of a controlled trial [6] shows that after daily intake of sugar-sweetened beverages over seven weeks, fasting FGF21 concentrations were increased in healthy men. This effect occurred in the glucose-, fructose- and sucrose-SSB intervention groups and thus was irrespective of the type of added sugar. Contrary to our assumption, fasting FGF21 concentrations were increased to a similar extent after consumption of glucose-, sucrose- and fructose-containing SSB, despite known substantial differences between the hexoses with respect to absorption, distribution and metabolism [34]. Although both fructose and glucose acutely induce a FGF21 serum response that is considered to result from stimulation of the sugar-ChREBP-FGF21 signaling axis in the liver, the acute FGF21 response induced by fructose was found to be stronger than the one induced by glucose [30]. This difference may be explained by the fact that fructose is a more potent hepatic ChREBP inducer than glucose because absorbed fructose is very rapidly and almost completely metabolized by the liver. The liver is considered the main site of fructose metabolism and the predominant source of circulating FGF21. However, thresholds of monosaccharide levels required for induction of the hepatic ChREBP-FGF21 axis may be achieved after consumption of both fructose- and glucose-sweetened beverages, and thus, the effects of regular consumption of glucose- and fructose-sweetened beverages regarding fasting FGF21 serum concentrations may be similar.

Furthermore, the liver is not the only site of FGF21 expression. The muscles and adipose tissue are additional sources of FGF21. Thus, it could be hypothesized that the relative contribution of the liver-, muscle- and fat-derived FGF21 to FGF21 serum concentrations may be different during fructose- vs. sucrose- vs. glucose-SSB interventions. It has been shown that insulin induces FGF21 expression in the muscles, which contributes to circulating FGF21 [35]. Therefore, the muscles may also represent a possible site of origin of circulating FGF21, in particular, after glucose consumption. Furthermore, there is evidence of leptin-induced FGF21 expression in WAT [36]. Notably, leptin levels were increased after the interventions with SSB containing glucose, which would be consistent with increased FGF21 levels. Increased FGF21 expression in adipose tissue after a high-glucose but not after high-fructose diet has been demonstrated in mice [37]. To our knowledge, the contribution of FGF21 expressed by muscles or adipose tissue (in response to glucose and fructose) to circulating FGF21 has not yet been investigated in humans. Future studies should investigate the effects of SSB consumption on gene expression of FGF21, as well as its receptors and target genes in different tissues (liver, muscle, brown adipose tissue (BAT) and WAT).

In the current study, as well as in previous studies [7], we observed no overall increase in energy intake by SSB interventions, but we did find an overall higher sugar intake by SSB interventions. Thus, SSB consumption did not lead to a fully compensatory reduction of sugar intake from other sources. However, we observed a reduction of the energy intake from protein and/or fat (i.e., in the glucose and fructose group), which is consistent with previous SSB studies [7]. This finding implies that sugar intake is not solely regulated by FGF21 but is subject to a regulatory network with additional players that still need to be identified.

Increased FGF21 expression could be regarded as an adaptive response that contributes to glucose homeostasis in individuals regularly exposed to sugar loads. FGF21 induces glucose disposal by different mechanisms and thereby maintains blood-sugar homeostasis. As an important FGF21 target tissue, adipose tissue increases adiponectin secretion, glucose uptake and uncoupling protein 1 (UCP1) expression [15]. We also measured adiponectin serum concentrations, as FGF21 is considered to increase adiponectin concentration [38], which then increases insulin sensitivity and promotes adipogenesis and glucose uptake by AT [38,39]. In the present study, we observed no changes in adiponectin concentrations. However, it remains open whether adiponectin expression in adipose tissue was increased by the intervention of the study.

Similarly, the levels of resistin (which was also shown to be involved in the adiponectin–FGF21 axis [40]) also remained unchanged.

A change was observed in C-peptide levels, with an increase in fasting C-peptide in the sucrose group possibly pointing to a slight decrease in insulin sensitivity after 7 weeks of consumption of sucrose-containing SSBs.

Assuming a state of FGF21 resistance in metabolic diseases, as described above, the question is whether this state can be reversed. Indeed, animal studies have been able to show that a reduction of obesity-induced metabolic disturbances via enhancing FGF21 sensitivity in adipose tissue is possible, e.g., by exercise [41]. It remains to be clarified whether such mechanisms also play a role in humans.

Our observation that regular SSB consumption may increase fasting FGF21 concentrations contrasts with a recent study reporting that fructose intake does not change baseline FGF21 concentrations in men [42]. This discrepancy may be explained by the different interventions in the studies. Subjects of the latter study had to consume a daily total dose of 75 g fructose according to their individual preference within 24 h (over two weeks). This means that they possibly ingested the fructose at single doses below the threshold of 20 g required to induce an FGF21 response [43]. This threshold was achieved with our study, in which subjects had to consume 80 g of sugar as SSB drinks divided into three doses per day.

Our study has some limitations. First, our analysis is a descriptive secondary report of data from a randomized controlled trial. Thus, it was not powered to detect between-group differences regarding FGF21 levels. Its character is exploratory, and thus, further trials are needed to confirm the findings. Since this would necessitate a group size of 60–90 participants as calculated using the actual variances observed in this study, it might be advisable to use a cross-over design to reduce interindividual differences. However, it must be taken into account that a cross-over design allowing the comparison of effects of different sugar types would impose a long-term obligation on study participants. Second, it is an inherent problem of dietary studies that awareness of being on a dietary study, per se, may impact the outcome, and that choosing an adequate placebo is difficult. A placebo group receiving artificially sweetened beverages was not used in the study, as such non-caloric sweeteners potentially affect human metabolism (e.g., appetite control, weight and microbiome composition) [44,45]. For future studies, a water control might by an option to at least control for the volume provided. Third, women were not included in our study, as divergent metabolic effects of fructose on men and women are known, which could confound the results [32]. However, the inclusion of young men can be justified, as they generally have a higher SSB intake and thus represent a population with a possibly increased risk to develop metabolic alterations [46]. Fourth, the study neither explored the origin of serum FGF21 after SSB consumption nor the transcriptional response in FGF21 target tissues. Of course, these measurements are technically demanding and require biopsies; they should be addressed in future studies.

## 5. Conclusions

This study describes an increase in fasting FGF21 concentrations after moderate daily SSB consumption as compared to a control group. Interestingly, effects of glucose-, fructose- and sucrose-sweetened beverages on FGF21 were comparable, indicating that repeated intake of SSB over the course of several weeks elevated FGF21 levels irrespective of the type of sugar consumed. Accordingly, SSB consumption may contribute to the emergence of FGF21 resistance that may affect metabolic health. Further studies are required to confirm our findings and to provide better understanding of the impact of SSB consumption on FGF21 physiology.

## Figures and Tables

**Table 1:** Anthropometric characteristics at baseline (W0) and after intervention (W7).

Variable		Control Group *N* = 21	Glucose Group *N* = 20	Fructose Group *N* = 23	Sucrose Group *N* = 19
Age (years)	W0	22.5 (21.2–23.8)	22.0 (20.8–23.1)	23.6 (22.6–24.5)	23.4 (22.3–24.4)
Body weight (kg)	W0	69.7 (65.8–73.7)	71.4 (68.0–74.7)	69.5 (65.8–73.1)	76.2 (72.9–79.4)
	W7	69.9 (66.0–73.8)	71.9 (68.7–75.1)	69.8 (66.2–73.4)	76.7 (73.6–79.9)
	Δ	0.0 (−0.9–1.4) ^1^	0.3 (−0.2–1.4) ^1^	0.8 (−0.7–1.2) ^1^	0.2 (−0.4–1.3) ^1^
Height (m)	W0	1.81 (1.78–1.84)	1.81 (1.78–1.84)	1.80 (1.77–1.83)	1.83 (1.80–1.86)
W/H ratio	W0	0.88 (0.87–0.90)	0.85 (0.83–0.87)	0.87 (0.85–0.89)	0.87 (0.85–0.89)
	W7	0.88 (0.87–0.90)	0.85 (0.83–0.87)	0.87 (0.85–0.89)	0.88 (0.86–0.90)
	Δ	0.00 (−0.01–0.02)	0.00 (−0.02–0.02)	0.00 (−0.02–0.01)	0.01 (−0.01–0.02)

Abbreviations: W/H, waist hip ratio. Data are presented as means (CI) or medians (25th and 75th percentile values) ^1^.

**Table 2:** Lipid and glucose metabolism at baseline (W0) and after intervention (W7).

Variable		Control Group *N* = 21	Glucose Group *N* = 20	Fructose Group *N* = 23	Sucrose Group *N* = 19
Fasting glucose (mmol/L)	W0	4.33 (3.97–4.63)	4.26 (4.09–4.42)	4.38 (4.15–4.53)	4.40 (4.20–4.66)
	W7	4.47 (4.09–4.67)	4.39 (4.09–4.56)	4.44 (4.16–4.73)	4.32 (4.24–4.57)
	Δ	0.03 (−0.16–0.23) ^1^	0.05 (−0.14–0.23) ^1^	0.01 (−0.15–0.17) ^1^	−0.02 (−0.14–0.10) ^1^
Insulin (pmol/L)	W0	104.1 (79.3–137.7)	98.3 (80.6–121.8)	103.4 (75.6–134.9)	94.7 (45.7–123.0)
	W7	90.9 (76.0–117.6)	107.4 (83.2–143.0)	99.3 (83.5–154.8)	89.8 (53.9–108.9)
	Δ	−13.0 (−34.6–8.6) ^1^	11.5 (−12.3–35.4) ^1^	−6.3 (−34.4–21.7) ^1^	−8.2 (−32.0–15.5) ^1^
c-peptide (pmol/L)	W0	410.0 (360.0–445.0)	435.0 (360.5–560.0)	390.0 (290.0–510.0)	350.0 (290.0–430.0)
	W7	380.0 (355.0–490.0)	480.0 (324.0–610.0)	390.0 (310.0–510.0)	430.0 (280.0–500.0)
	Δ	−13.6 (−68.8–41.6) ^1^	−11.7 (−72.4–49.1) ^1^	−3.7 (−38.4–31.0) ^1^	55.8 (6.0–105.6) ^1^
Triglycerides (mmol/L)	W0	0.77 (0.66–0.93)	0.72 (0.53–0.88)	0.84 (0.58–1.15)	0.84 (0.61–0.92)
	W7	0.74 (0.62–0.94)	0.72 (0.53–1.12)	0.86 (0.61–1.04)	0.68 (0.58–0.88)
	Δ	−0.00 (−0.21–0.14)	0.03 (−0.09–0.27)	−0.08 (−0.31–0.07)	−0.12 (−0.27–0.13)
FFA (µmol/L)	W0	428 (361–700)	531 (463–770)	520 (340–703)	464 (343–745)
	W7	481 (393–580)	368 (284–547)	363 (296–472)	441 (273–754)
	Δ	30 (−269–114)	−172 (−363–51)	−117 (−263–28)	−87 (−407–187)
Total cholesterol (mmol/L)	W0	3.80 (3.40–4.35)	3.60 (3.30–4.30)	3.90 (3.60–4.50)	4.10 (3.40–4.50)
	W7	3.70 (3.25–4.25)	3.80 (3.20–4.35)	3.80 (3.60–4.10)	3.9 (3.58–4.35)
	Δ	−0.10 (−0.30–0.00)	0.00 (−0.35–0.40)	−0.10 (−0.50–0.10)	0.10 (−0.50–0.30)
LDL cholesterol (mmol/L)	W0	2.00 (1.55–2.55)	1.80 (1.50–2.30)	2.35 (1.90–2.68)	2.20 (1.70–2.70)
	W7	2.00 (1.45–2.50)	1.95 (1.40–2.38)	2.00 (1.80–2.60)	2.10 (1.65–2.53)
	Δ	0.00 (−0.15- 0.10)	−0.10 (−0.20–0.30)	−0.10 (−0.50–0.20)	0.00 (−0.40–0.20)
HDL cholesterol (mmol/L)	W0	1.39 (1.25–1.62)	1.45 (1.27–1.70)	1.36 (1.09–1.55)	1.40 0 (1.16–1.71)
	W7	1.37 (1.11–1.51)	1.48 (1.15–1.67)	1.29 (1.13–1.62)	1.49 (1.28–1.67)
	Δ	−0.07 (−0.21–−0.01)	−0.05 (−0.10–0.10)	0.02 (−0.18–0.14)	0.08 (−0.12–0.16)
Leptin (ng/mL)	W0	2.1 (1.1–3.4)	2.0 (0.8–3.1)	2.4 (0.7–3.3)	2.7 (1.4–3.3)
	W7	2.1 (1.1–3.1)	2.1 (1.4–4.0)	2.6 (0.9–3.5)	3.2 (1.7–4.3)
	Δ	0.1 (−0.5–0.6) ^1^	0.6 (−0.2–1.4) ^1^	0.3 (−0.1–0.6) ^1^	0.6 (0.1–1.1) ^1^
Resistin (ng/mL)	W0	3.6 (3.0–4.3)	3.9 (3.2–4.5)	4.1 (3.1–5.0)	3.7 (3.1–4.2)
	W7	3.7 (2.8–4.3)	3.8 (3.2–5.0)	4.0 (3.2–4.5)	3.5 (3.1–4.3)
	Δ	0.1 (−0.4–0.2)	0.0 (−0.3–0.7)	−0.3 (−0.6–0.1)	0.1 (−0.3–0.4)
Adiponectin (µg/mL)	W0	6.0 (4.2–9.2)	6.0 (3.7–6.9)	5.1 (3.7–8.7)	4.9 (3.6–7.0)
	W7	5.8 (3.9–9.1)	6.2 (4.5–7.2)	4.6 (3.5–7.4)	5.6 (4.3–6.7)
	Δ	−0.2 (−1.0–0.7)	0.4 (−0.6–1.6)	−0.3 (−1.1–0.3)	0.1 (−0.7–0.8)
FGF21 (pg/mL)	W0	49.3 (18.6–81.0)	51.6 (42.1–65.6)	45.0 (24.5–82.5)	31.6 (20.4–59.0)
	W7	61.4 (33.6–90.3)	70.3 (26.6–107.1)	53.9 (38.9–108.8)	58.6 (32.0–102.8)
	Δ	6.3 (−20.1–26.9)	17.4 (0.4–45.8)	22.9 (−8.6–35.1)	13.7 (2.2–46.1)

Abbreviations: FGF, fibroblast growth factor; LDL, low-density lipoprotein; HDL, high-density lipoprotein. Data are presented as medians (25th and 75th percentile values) or means (CI) ^1^.

**Table 3:** Dietary intake of the subjects of the different groups at baseline (W0) and after intervention (W7).

		Control *N* = 21	Glucose *N* = 18	Fructose *N* = 19	Sucrose *N* = 18
Energy (kcal/day)	W0	2085 (1887–2662)	2024 (1791–2462)	1835 (1502–2322)	2214 (1976–2723)
	W7	1898 (1687–2364)	2389 (1876–2510)	2131 (1707–2574)	1894 (1640–2911)
	Δ	−187 (−396–−36)	112 (−234–497)	155 (−223–578)	−68 (−936–311)
Carbohydrate, %	W0	47 (44–49) ^2^	47 (44–50) ^2^	47 (43–50) ^2^	47 (44–50) ^2^
	W7	48 (44–52) ^2^	58 (55–61) ^2^	56 (53–60) ^2^	52 (48–56) ^2^
	Δ	1 (−2–5) ^2^	11 (7–15) ^2^	10 (5–14) ^2^	4 (0–8) ^2^
Protein, %	W0	17 (15–18) ^2^	18 (15–20) ^2^	17 (15–19) ^2^	19 (17–21) ^2^
	W7	18 (16–19) ^2^	15 (14–17) ^2^	14 (13–15) ^2^	17 (15–19) ^2^
	Δ	1 (−1–3) ^2^	−3 (−5–0) ^2^	−2 (−5–0) ^2^	−2 (−4–1) ^2^
Fat, %	W0	37 (35–39) ^2^	35 (32–38) ^2^	36 (32–39) ^2^	35 (32–38) ^2^
	W7	35 (32–38) ^2^	29 (26–32) ^2^	29 (26–32) ^2^	33 (29–37) ^2^
	Δ	−3 (−8–2)	−4 (−11–−1)	−7 (−10–−2)	−2 (−6–0)
Fructose ^1^, g/d	W0	6.9 (4.3–15.4)	4.5 (1.2–7.9)	5.8 (2.8–9.8)	7.8 (1.9–12.4)
	W7	5.1 (2.2–7.3)	3.6 (1.8–6.8)	81.8 (79.7–86.1)	2.8 (0.6–7.3)
	Δ	−1.8 (−9.3–1.0)	−0.5 (−3.8–1.1)	75.1 (63.7–80.6)	−4.4 (−8.8–0.1)
Glucose ^1^, g/d	W0	4.8 (2.8–10.8)	4.4 (1.4–8.1)	5.3 (2.1–8.6)	5.9 (2.1–8.9)
	W7	5.0 (2.1–6.0)	83.3 (80.6–84.2)	3.5 (2.1–6.8)	2.6 (0.7–5.5)
	Δ	−0.5 (−6.7–1.3)	78.0 (75.6–79.7)	−2.0 (−4.9–1.8)	−0.7 (−6.7–0.6)
Sucrose, g/d	W0	61.1 (43.1–81.9)	50.2 (32.9–83.6)	43.2 (32.2–82.5)	78.6 (56.4–94.8)
	W7	51.0 (33.6–70.9)	48.9 (28.8–68.0)	51.8 (27.7–65.4)	118.1 (101.9–146.0)
	Δ	−10.8 (−23.5–2.0) ^2^	−5.4 (−18.0–7.1) ^2^	1.9 (−15.1–18.9) ^2^	45.2 (25.4–65.1) ^2^

^1^ as monosaccharide. Values are shown as medians (25th and 75th percentile values) or means (CI) ^2^.

## Data Availability

The data presented in this study are available on request from the corresponding author.
